# The Phonological Mapping (Mismatch) Negativity: History, Inconsistency, and Future Direction

**DOI:** 10.3389/fpsyg.2020.01967

**Published:** 2020-08-25

**Authors:** Jennifer Lewendon, Laurie Mortimore, Ciara Egan

**Affiliations:** ^1^School of Languages, Literatures and Linguistics, Prifysgol Bangor University, Bangor, United Kingdom; ^2^School of Psychology, Prifysgol Bangor University, Bangor, United Kingdom; ^3^Department of Experimental Psychology, University of Oxford, Oxford, United Kingdom

**Keywords:** event-related potentials, phonology, PMN, N400, MMN, phonological mismatch, phonological mapping, language

## Introduction

The last three decades have seen a considerable growth in the use of event-related potential (ERP) methods in language research. As our appreciation of the cognitive mechanisms underlying language processing increases, so too does our understanding of its electrophysiological correlates. The phonological mapping negativity^1^ (PMN) is an ERP component long established to index pre-lexical phonological processing (Connolly and Phillips, 1994; Connolly et al., 2001; Desroches et al., 2009), involving the mapping of speech signals onto phonological representations (Newman and Connolly, 2009). Generally maximal at around 300 ms post stimulus onset (PSO), the PMN is classically elicited in paradigms in which phonological expectancies are generated using words (Newman and Connolly, 2009), sentences with high cloze probability (Connolly and Phillips, 1994), and pictures (Desroches et al., 2009). For example, asking participants to delete the initial sound from a word (e.g., snap without the /s/) will generate expectation of “nap.” When this expectation of particular phonological input is violated (e.g., presentation of “tap” in place of “nap”) the component increases in amplitude.

ERP components are typically identified through a combination of their scalp distribution, timing, polarity, and sensitivity to experimental manipulations (Woodman, 2010). Marked overlaps in timing and sensitivity with another component, the N400, initially prompted research intended to distinguish the PMN as a discrete response (Connolly et al., 1992; Connolly and Phillips, 1994). Over the last 30 years however, such research has unveiled a host of inconsistencies in the reported topography, timing, and sensitivity of the response. Despite its established place in the core repertoire of ERP components as a reliable, well-defined effect, consistent ambiguity in the characterization of the response necessitates reconsideration of the reliability and authenticity of the PMN.

## Distinguishing the PMN & N400

The N400 is the most commonly studied electrophysiological response in the context of language processing, sensitive to the manipulation of semantic, phonological, and associative relationships (Kutas and Federmeier, 2011). Notably, the N400 can be modulated by phonological factors such as word-initial and word-final overlap (Bölte and Coenen, 2002; O'Rourke and Holcomb, 2002). The component's breadth of sensitivity means that it is generally characterized as a function of its timing, behavior and morphology, with the term N400 used as a heuristic label for stimulus related brain activity that occurs in a pattern of sensitivity to manipulated variables 200–600 ms post-stimulus-onset (Kutas and Federmeier, 2011).

The degree to which the PMN and N400 overlap, or coexist as functionally distinct components, has been the subject of substantial debate. Early PMN literature focused on the differentiation of these components by highlighting the sensitivity of the PMN to phonological violations, and that of the N400 to semantic violations. In one of the first studies to report a dissociation between the N400 and PMN, Connolly and Phillips (1994) sought to distinguish the effects of phonological and semantic violations by manipulating both factors in high cloze-probability sentences. Target words were fully congruent (e.g., The piano was out of **tune**); semantically congruent but phonologically unexpected [e.g., The pig wallowed in the **pen** [mud]]; semantically unexpected but phonologically congruent [e.g., The gambler had a streak of bad **luggage** [luck]]; or fully incongruent [e.g., Joan fed her baby some warm **nose** [food]]. Whilst a combined PMN–N400 response was elicited in the fully incongruent condition, pure phonological violations elicited a PMN response, and pure semantic violations elicited a slightly later N400 modulation. The authors interpreted the results as indicating that the two components were functionally distinct, with the PMN sensitive to early lexical processing, and the N400 representing later semantic integration processes. Although oft referenced as a defining study on the PMN, further consideration suggests that such an interpretation may be oversimplified. Firstly, the largest PMN modulation was found in response to fully incongruent targets, as opposed to phonological mismatch alone. Furthermore, when only semantic expectation was violated, N400 peak latency was delayed by over 50 ms relative to the fully incongruent condition. Both the heightened PMN response to fully incongruent stimuli, suggesting sensitivity to both phonological and semantic manipulation, as well as the increased N400 latency for purely semantic violations suggest the two components may not be as functionally independent as proposed.

Another significant study in the characterization of the PMN was Connolly et al. (2001), which investigated the sensitivity of the component to semantic manipulations. Here, the PMN was unaffected by lexicality—a result that was interpreted to suggest that unlike the N400, the PMN does not reflect semantic processing. Despite the prevailing influence of the study, conflicting MEG evidence (Kujala et al., 2004) subsequently led to the assertion that no definitive conclusions about the PMN could be drawn from the paradigm used (Newman and Connolly, 2009). In a later investigation into whether the PMN reflects pre-lexical or lexical processing, Newman et al. (2003) presented participants with auditory prime words, and asked them to mentally delete the initial consonant (e.g., *clap*, /k/). Subsequently, participants either heard a correct target (e.g., *lap*) or one that violated expectation in one of three ways, (i) wrong consonant deletion (WC, e.g., *cap*); (ii) consonant cluster deletion (CC, e.g., *ap*); or (iii) an irrelevant word (IW, e.g., *nose*). The authors predicted gradations in response by phonological violation (largest in IW condition, followed by WC and CC), and an indistinguishable response to word and non-word targets demonstrating the PMN's insensitivity to lexicality. Results showed that PMN mean amplitude was significantly reduced in the correct condition, whilst it was significantly greater and undistinguishable across all incorrect conditions. These findings were interpreted as evidence for the PMN's phonological sensitivity and functional distinction from the N400 based on its comparable amplitude for both word and non-words targets. Despite their interpretation, the authors acknowledged the likelihood that P300 contamination uniquely reduced PMN amplitude in the correct condition. Such contamination would likely confound any comparison between correct and incorrect conditions. As such, in the three remaining conditions with reliable results (IW, WC, & CC), PMN amplitude failed to differ significantly despite the predictions of gradations in response to phonological violation. Furthermore, the absence of N400 reporting weakens the interpretation significantly, as it is not possible to determine whether the study's task demands would have elicited N400 lexicality distinctions. Without evidence of a lexicality-dependant response from a component known to be sensitive to semantics, the absence of a PMN lexicality response cannot be deemed meaningful. A similar later study, wherein participants were asked to delete the initial consonant from four-syllable words, reported a PMN response to correct/incorrect targets and a lexicality effect on an N400-like response (Newman and Connolly, 2009). Although the two components were concluded to be functionally dissociated, visual inspection revealed a larger PMN response to words compared to non-words over centroparietal sites, which the authors attributed to the onset of the N400.

Despite their methodological issues, current literature predominantly defines PMN based on the aforementioned studies. Furthermore, Table 1 shows wider research often cited when defining the component, noting further methodological issues including low sample size, known to affect the noise to signal ratio of ERP data (Boudewyn et al., 2018). Whilst literature supporting the specific PMN component as reported by Connolly and Phillips (1994) appears conflicting, there remains consistent evidence that ERP activity between 200 and 400 ms PSO reflects the activation and processing of phonological information (Hagoort and Brown, 2000; van den Brink et al., 2001; Desroches et al., 2009). Alternative explanations for phonological sensitivity within this timeframe range from early N400 accounts (Praamstra et al., 1994; Van Petten et al., 1999; Dumay et al., 2001); separate components including the P250 and P325 (Hagoort and Brown, 2000; van den Brink et al., 2001; Holcomb and Grainger, 2006; Grainger and Holcomb, 2009); to variations upon a visual PMN component (Desroches et al., 2009; Sučević et al., 2015).

**Table 1:** Summary of key studies charaterizing the PMN, reported effect topographies and methodological considerations.

**References**	**Topography**	**Methodological considerations**
Connolly et al. (1990)	Unsubtracted waves: frontocentral; Subtracted (difference) waves: central	10 participants (trials per condition unclear).
Connolly et al. (1992)	Flat distribution across midline sites	Response not visible in averaged waveforms.
Connolly and Phillips (1994)	Frontal, central, and parietal	^*^
Van Petten et al. (1999)	Flat distribution across scalp	
D'Arcy et al. (2000)	Early N2b (130–230 ms): parietal	
Connolly et al. (2001)	Frontal	10 participants (min. 60 trials per condition). Conflicting MEG data acknowledged to invalidate PMN results.^*^
Hagoort and Brown (2000)	Exp. 1: posterior Exp. 2: no interaction with site.	12 participants (60 trials per condition). “N200” response to semantic expectation violations. No isolation of phonological anomaly.
van den Brink et al. (2001)	Flat distribution across scalp	
Newman et al. (2003)	Frontotemporal	Early onset P300 contamination in phonological expected condition. Authors could not confirm absence of PMN in this condition.^*^
D'Arcy et al. (2004)	Frontocentral	10 participants (24 trials per condition).
Newman and Connolly (2009)	Frontal and central	13 participants (40 trials per condition).^*^

* *See text for full discussion of methodological limitations*.

## A Question of Topography?

Methodological discrepancies in the aforementioned research maintain the possibility that central/parietal PMN responses may not be as distinct from the N400 as generally assumed. This does, prompt questions as to the origin of the frontal PMN responses. Despite considerable variability in reported PMN topography (see Table 1), a number of studies reporting frontocentral PMN topography have influenced the notion that the component is classically anterior (Connolly et al., 1990; D'Arcy et al., 2004).

The frontocentral topography reported by these studies is not too dissimilar from that of another ERP response, the auditory MMN. Originally reported by Näätänen et al. (1978), the MMN is a frontocentral component that peaks ~150–250 ms PSO (Kujala et al., 2007), and is typically elicited in oddball paradigms where it responds to infrequent deviant sounds presented amongst frequent “standard” sounds. Research suggests, however, that the MMN's sensitivity extends beyond pure auditory mismatch. For example, Shtyrov et al. (2003) presented inattentive participants with short phrases that differed in a single phoneme, rendering them either grammatical or ungrammatical. The authors found that infrequent ungrammatical phrases elicited an MMN amplitude increase. Further research, suggesting the MMN may be sensitive to semantic (Pulvermüller et al., 2001), acoustic, and phonological properties of speech (Aaltonen et al., 1997; Weber et al., 2004) imply that the component may be more diverse in sensitivity than originally thought.

Despite considerable overlap in both timing and topography, in addition to recent reports of a potential phonological Mismatch Negativity response (Proverbio et al., 2018), no study has thus far compared the PMN source loci with those of the MMN (Näätänen et al., 2007). Whilst the PMN requires active participant attention, the MMN is pre-attentive. It has been assumed thus far that the necessity of attentiveness means the two components are generated by distinct cortical mechanisms (Näätänen et al., 2007). Perhaps however, the mere generation of phonological violation requires attention to linguistic content in order to establish expectation, whilst simple auditory mismatches, such as those typical in MMN paradigms, do not. If so, the MMN and frontal PMN could instead represent two ends of a spectrum of electrophysiological responses to auditory mismatch, the former as a rudimentary reaction to auditory mismatch, and the latter a more sophisticated response to violation of complex expectations. Whilst establishing the extent of the distinction between the two components is beyond the scope of this article, the necessity for future work to ascertain the discreteness of these two components beyond mere assumption is crucial (Kujala et al., 2007).

## Concluding Remarks and Future Perspectives

The PMN has consistently been reported as a distinct electrophysiological index of phonological processing. In the present article, we highlight the necessity to reconsider the clear distinctions drawn by prior research between the PMN, N400, and MMN. We propose that the frontal PMN response cannot be differentiated from the MMN based on attentional processing requirements alone. Furthermore, despite the continued prominence of research discussed, we suggest that the PMN response cannot be sufficiently differentiated from the N400 based on current research. Instead, it remains possible that the centroparietal PMN may represent earlier onset of congruity effects in response to phonemic mismatch with an expected word (Van Petten et al., 1999).

Future research in this field must focus on elucidating inconsistencies in topography and sensitivity of the PMN in order to gain a thorough understanding of this component. This may be achieved through several means: (1) Utilizing cluster-based permutation approaches as a data-driven way to choose time-windows and electrodes for analysis, this could clarify the results of experimental paradigms where both a PMN and N400 appear to be present. (2) Conducting a meta-analysis of the PMN literature to gain clarity regarding current evidence on the PMN; and (3) Conducting further research utilizing paradigms intended to dissociate the PMN from the N400 and MMN. Until further evidence becomes available, it is clear that a full appreciation of the insight ERPs can offer us into phonological processing remains beyond our reach.

## Author Contributions

JL developed the initial concept of the field challenge. All authors listed have made a substantial, direct and intellectual contribution to the work, and approved it for publication.

## Conflict of Interest

The authors declare that the research was conducted in the absence of any commercial or financial relationships that could be construed as a potential conflict of interest.

## Footnotes

^1^Originally called the phonological mismatch negativity, the PMN has been relabeled the phonological mapping negativity both to distinguish it from the mismatch negativity, and to better describe its sensitivity not only to phonological mismatch, but fulfilled expectations.

## References

[B1] Aaltonen O. Eerola O. Hellström A. Uusipaikka E. Lang A. H. (1997). Perceptual magnet effect in the light of behavioral and psychophysiological data. J. Acoust. Soc. Am. 101, 1090–1105. 10.1121/1.418031 9035400

[B2] Bölte J. Coenen E. (2002). Is phonological information mapped onto semantic information in a one-to-one manner? Brain Lang. 81, 384–397. 10.1006/brln.2001.2532 12081407

[B3] Boudewyn M. Luck S. J. Farrens J. L. Kappenman E. S. (2018). How many trials does it take to get a significant ERP effect? It depends. Psychophysiology 55:e13049. 10.1111/psyp.13049 29266241 PMC5940511

[B4] Connolly J. F. Phillips N. A. (1994). Event-related potential components reflect phonological and semantic processing of the terminal word of spoken sentences. J. Cogn. Neurosci. 6, 256–266. 10.1162/jocn.1994.6.3.256 23964975

[B5] Connolly J. F. Phillips N. A. Stewart S. H. Brake W. G. (1992). Event-related potential sensitivity to acoustic and semantic properties of terminal words in sentences. Brain Lang. 43, 1–18. 10.1016/0093-934X(92)90018-A 1643505

[B6] Connolly J. F. Service E. D'Arcy R. C. N. Kujala A. Alho K. (2001). Phonological aspects of word recognition as revealed by high-resolution spatio-temporal brain mapping. NeuroReport 12, 237–243. 10.1097/00001756-200102120-00012 11209927

[B7] Connolly J. F. Stewart S. H. Phillips N. A. (1990). The effects of processing requirements on neurophysiological responses to spoken sentences. Brain Lang. 39, 302–318. 10.1016/0093-934X(90)90016-A 2224497

[B8] D'Arcy R. C. N. Connolly J. F. Crocker S. F. (2000). Latency shifts in the N2b component track phonological deviations in spoken words. Clin. Neurophysiol. 111, 40–44. 10.1016/S1388-2457(99)00210-2 10656509

[B9] D'Arcy R. C. N. Connolly J. F. Service E. Hawco C. S. Houlihan M. E. (2004). Separating phonological and semantic processing in auditory sentence processing: a high-resolution event-related brain potential study. Hum. Brain Mapp. 22, 40–51. 10.1002/hbm.20008 15083525 PMC6872076

[B10] Desroches A. S. Newman R. L. Joanisse M. F. (2009). Investigating the time course of spoken word recognition: electrophysiological evidence for the influences of phonological similarity. J. Cogn. Neurosci. 21, 1893–1906. 10.1162/jocn.2008.21142 18855555 PMC3965566

[B11] Dumay N. Benraïss A. Barriol B. Colin C. Radeau M. Besson M. (2001). Behavioral and electrophysiological study of phonological priming between bisyllabic spoken words. J. Cogn. Neurosci. 13, 121–143. 10.1162/089892901564117 11224913

[B12] Grainger J. Holcomb P. J. (2009). Watching the word go by: on the time-course of component processes in visual word recognition. Lang. Linguist. Compass. 3, 128–156. 10.1111/j.1749-818X.2008.00121.x 19750025 PMC2740997

[B13] Hagoort P. Brown C. M. (2000). ERP effects of listening to speech: semantic ERP effects. Neuropsychologia 38, 1518–1530. 10.1016/S0028-3932(00)00052-X 10906377

[B14] Holcomb P. J. Grainger J. (2006). On the time course of visual word recognition: an event-related potential investigation using masked repetition priming. J. Cogn. Neurosci. 18, 1631–1643. 10.1162/jocn.2006.18.10.1631 17014368 PMC1808538

[B15] Kujala A. Alho K. Service E. Ilmoniemi R. J. Connolly J. F. (2004). Activation in the anterior left auditory cortex associated with phonological analysis of speech input: localization of the phonological mismatch negativity response with MEG. Cogn. Brain Res. 21, 106–113. 10.1016/j.cogbrainres.2004.05.011 15325418

[B16] Kujala T. Tervaniemi M. Schröger E. (2007). The mismatch negativity in cognitive and clinical neuroscience: theoretical and methodological considerations. Biol. Psychol. 74, 1–19. 10.1016/j.biopsycho.2006.06.001 16844278

[B17] Kutas M. Federmeier K. D. (2011). Thirty years and counting: Finding meaning in the N400 component of the event related brain potential (ERP). Annu. Rev. Psychol. 62, 621–647. 10.1146/annurev.psych.093008.131123 20809790 PMC4052444

[B18] Näätänen R. Gaillard A. W. K. Mäntysalo S. (1978). Early selective-attention effect on evoked potential reinterpreted. Acta Psychol. 42, 313–329. 10.1016/0001-6918(78)90006-9 685709

[B19] Näätänen R. Paavilainen P. Rinne T. Alho K. (2007). The mismatch negativity (MMN) in basic research of central auditory processing: a review. Clin. Neurophysiol. 118, 2544–2590. 10.1016/j.clinph.2007.04.026 17931964

[B20] Newman R. L. Connolly J. F. (2009). Electrophysiological markers of pre-lexical speech processing: Evidence for bottom–up and top–down effects on spoken word processing. Biol. Psychol. 80, 114–121. 10.1016/j.biopsycho.2008.04.008 18524453

[B21] Newman R. L. Connolly J. F. Service E. Mcivor K. (2003). Influence of phonological expectations during a phoneme deletion task: evidence from event-related brain potentials. Psychophysiology 40, 640–647. 10.1111/1469-8986.00065 14570171

[B22] O'Rourke T. B. Holcomb P. J. (2002). Electrophysiological evidence for the efficiency of spoken word processing. Biol. Psychol. 60, 121–150. 10.1016/S0301-0511(02)00045-5 12270588

[B23] Praamstra P. Meyer A. S. Levelt W. J. M. (1994). Neurophysiological manifestations of phonological processing: latency variation of a negative ERP component timelocked to phonological mismatch. J. Cogn. Neurosci. 6, 204–219. 10.1162/jocn.1994.6.3.204 23964972

[B24] Proverbio A. M. Raso G. Zani A. (2018). Electrophysiological indexes of incongruent audiovisual phonemic processing: unraveling the McGurk effect. Neuroscience 385, 215–226. 10.1016/j.neuroscience.2018.06.021 29932985

[B25] Pulvermüller F. Kujala T. Shtyrov Y. Simola J. Tiitinen H. Alku P.. (2001). Memory traces for words as revealed by the mismatch negativity. NeuroImage 14, 607–616. 10.1006/nimg.2001.0864 11506534

[B26] Shtyrov Y. Pulvermüller F. Näätänen R. Ilmoniemi R. J. (2003). Grammar processing outside the focus of attention: an MEG Study. J. Cogn. Neurosci. 15, 1195–1206. 10.1162/089892903322598148 14709236

[B27] Sučević J. Savić A. M. Popović M. B. Styles S. J. Ković V. (2015). Balloons and bavoons versus spikes and shikes: ERPs reveal shared neural processes for shape–sound-meaning congruence in words, and shape–sound congruence in pseudowords. Brain Lang. 145–146, 11–22. 10.1016/j.bandl.2015.03.011 25935826

[B28] van den Brink D. Brown C. M. Hagoort P. (2001). Electrophysiological evidence for early contextual influences during spoken-word recognition: N200 versus N400 effects. J. Cogn. Neurosci. 13, 967–985. 10.1162/089892901753165872 11595099

[B29] Van Petten C. Coulson S. Rubin S. Plante E. Parks M. (1999). Time course of word identification and semantic integration in spoken language. J. Exp. Psychol. Learn. Memory Cogn. 25, 394–417. 10.1037/0278-7393.25.2.394 10093207

[B30] Weber C. Hahne A. Friedrich M. Friederici A. D. (2004). Discrimination of word stress in early infant perception: electrophysiological evidence. Brain Res. Cogn. Brain Res. 18, 149–161. 10.1016/j.cogbrainres.2003.10.001 14736574

[B31] Woodman G. (2010). A brief introduction to the use of event-related potentials (ERPs) in studies of perception and attention. Attention Perception Psychophys. 72, 2031–2046. 10.3758/APP.72.8.2031 21097848 PMC3816929

