# Corrigendum: The Phonological Mapping (Mismatch) Negativity: History, Inconsistency, and Future Direction

**DOI:** 10.3389/fpsyg.2020.619241

**Published:** 2021-01-28

**Authors:** Jennifer Lewendon, Laurie Mortimore, Ciara Egan

**Affiliations:** ^1^School of Languages, Literatures and Linguistics, Prifysgol Bangor University, Bangor, United Kingdom; ^2^School of Psychology, Prifysgol Bangor University, Bangor, United Kingdom; ^3^Department of Experimental Psychology, University of Oxford, Oxford, United Kingdom

**Keywords:** event-related potentials, phonology, PMN, N400, MMN, phonological mismatch, phonological mapping, language

In the original article, there were the following mistakes in Table 1 as published:

- Row 2, column 2. “substraced” replaced with “subtracted.”
- Row 4, column 3. Information about incorrect study. Information deleted.
- Row 5. Column 2. “midline” amended to “scalp”
- Row 6. Column 2. “Late N2b (250–350 ms): distributed across scalp” deleted – irrelevant.
- Row 7: Column 2. Updated to reflect the dual-study results.

**Table 1:** Summary of key studies charaterizing the PMN, reported effect topographies and methodological considerations.

**References**	**Topography**	**Methodological considerations**
Connolly et al. (1990)	Unsubtracted waves: frontocentral; Subtracted (difference) waves: central	10 participants (trials per condition unclear).
Connolly et al. (1992)	Flat distribution across midline sites	Response not visible in averaged waveforms.
Connolly and Phillips (1994)	Frontal, central, and parietal	^*^
Van Petten et al. (1999)	Flat distribution across scalp	
D'Arcy et al. (2000)	Early N2b (130–230 ms): parietal	
Connolly et al. (2001)	Frontal	10 participants (min. 60 trials per condition). Conflicting MEG data acknowledged to invalidate PMN results.^*^
Hagoort and Brown (2000)	Exp. 1: posterior Exp. 2: no interaction with site.	12 participants (60 trials per condition). “N200” response to semantic expectation violations. No isolation of phonological anomaly.
van den Brink et al. (2001)	Flat distribution across scalp	
Newman et al. (2003)	Frontotemporal	Early onset P300 contamination in phonological expected condition. Authors could not confirm absence of PMN in this condition.^*^
D'Arcy et al. (2004)	Frontocentral	10 participants (24 trials per condition).
Newman and Connolly (2009)	Frontal and central	13 participants (40 trials per condition).^*^

* *See text for full discussion of methodological limitations*.

The corrected Table 1 appears below.

**Corrections have also been made to the title and keywords of the original article**.

The corrected title and keywords are shown below:

Title: The Phonological Mapping (Mismatch) Negativity: History, Inconsistency, and Future Direction.

keywords: event-related potentials, phonology, PMN, N400, MMN, phonological mismatch, phonological mapping, language.

The authors apologize for these errors and state that they do not change the scientific conclusions of the article in any way. The original article has been updated.
